# Supplementary material for: Pharmacological Inhibition of the VCP/Proteasome Axis Rescues Photoreceptor Degeneration in RHOP23H Rat Retinal Explants
Source: Biomolecules. 2021 Oct 16;11(10):1528. doi: 10.3390/biom11101528 (PMC8534135; doi:10.3390/biom11101528)
Supplement: Supplementary file 1 [file biomolecules-11-01528-s001.zip › biomolecules-1360836-supplementary.pdf]

Supplementary file

Pharmacological inhibition of the VCP/proteasome axis rescues photoreceptor degeneration in RHO^{P23H} rat retinal explants

Merve Sen, Oksana Kutsyr, Bowen Cao, Sylvia Bolz, Blanca Arango-Gonzalez*, Marius Ueffing*

Table S1: References list for the chosen concentrations in this study.

Treatment	Concentrations	References
Geldanamycin	0.01, 0.1, and 1 µM	Wu et al., 2010; Karkoulis et al., 2013
Kifunensine	1, 10 and 100 µM	Kosmaoglu et al., 2009; Saeed et al., 2011; Elfrink et al., 2013
NMS-873	0.5, 1 and 5 µM	Lin et al., 2017; Bastola et al., 2017
Bortezomib	0.01, 0.1, and 1 µM	Hili et al., 2009, Obeng et al., 2006

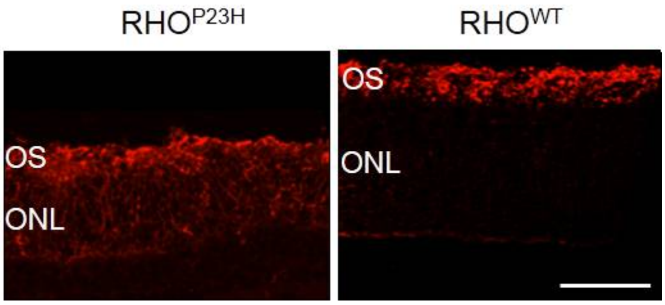

Figure S1: Rhodopsin localization in RHO^{P23H} and RHO^{WT} retinal organotypic cultures. Fluorescent labeling in cryosections designates the location of rhodopsin (red staining) in RHO^{P23H} and RHO^{WT} organotypic retinal cultures at PN15, explanted at postnatal day 9 and cultivated for 6 days (PN9 DIV6). Scale bar is 50 µm. OS: outer segment, ONL: outer nuclear layer, RHO: rhodopsin, WT: wild type.

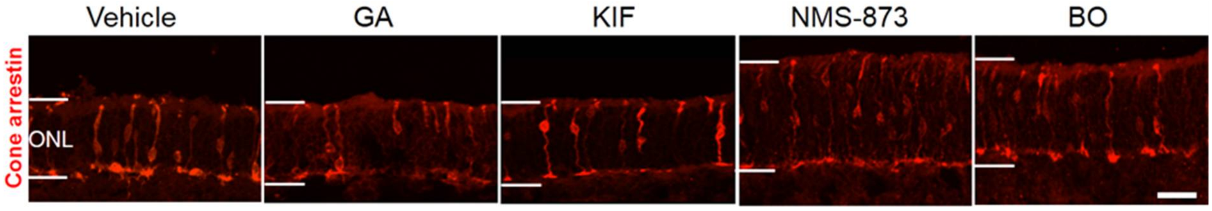

Figure S2: Pharmacological interference in the VCP/proteasome axis increases the organization of cone photoreceptors in RHO^{P23H} retinal organotypic cultures. Fluorescent labeling in cryosections designates the location of cone arrestin (red staining) in RHO^{P23H} retinae organotypic cultures at PN15, explanted at postnatal day 9 and cultivated for 6 days (PN9 DIV6), treated with corresponding vehicles (H_2O or DMSO) or Hsp90 inhibitor GA (1 µM), ERM1 inhibitor KIF (100 µM), VCP inhibitor NMS-873 (5 µM), and proteasome inhibitor BO (1 µM). Retinae treated with VCP or proteasome inhibitors but not with GA or KIF showed increased cone length and migration to the ONL. Scale bar is 50 µm. ONL: outer nuclear layer, GA: Geldanamycin, KIF: Kifunensine, BO: Bortezomib.

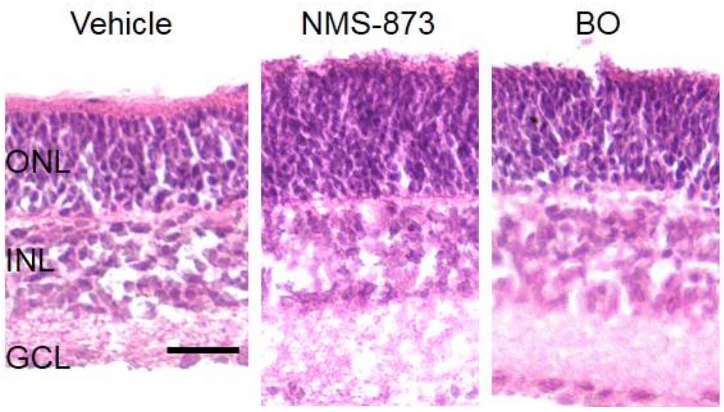

Figure S3: Modulation of the VCP/proteasome axis improves retinal structure in RHO^{P23H} retinal organotypic cultures. Retinae of RHO^{P23H} transgenic rats were explanted at postnatal day 9 and cultivated for 6 days (PN9 DIV6), treated with corresponding vehicle (DMSO) or VCP inhibitor NMS-873 (5 μM), and proteasome inhibitor BO (1 μM). Scale bar is 20 μm. ONL: outer nuclear layer, INL: inner nuclear layer, GCL: ganglion cell layer, BO: Bortezomib.

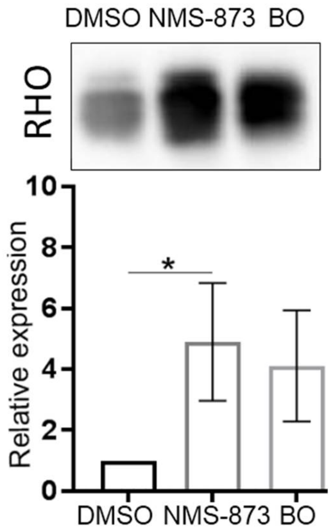

Figure S4: Representative image of Western blot detection of rhodopsin for the lysate of RHO^{P23H} organotypic retinal explants at PN15, explanted at postnatal day 9 and cultivated for 6 days (PN9 DIV6), treated with DMSO (1%), NMS-873 (5 μM), and BO (1 μM). Densitometric analysis of RHO band intensities (37 kDa) in the treated retinae to the vehicle treated control showed increased RHO expression after inhibition of VCP or proteasome in RHO^{P23H} retinae. The data are presented as mean ±SD, and one-way ANOVA analysis was performed. *$p<0.05$. RHO: rhodopsin, BO: Bortezomib.

References:

Wu, W.C.; Wu, M.H.; Chang, Y.C.; Hsieh, M.C.; Wu, H.J.; Cheng, K.C.; Lai, Y.H.; Kao, Y.H. Geldanamycin and its analog induce cytotoxicity in cultured human retinal pigment epithelial cells. Exp. Eye Res. 2010, 91, 211–219, doi:10.1016/j.exer.2010.05.005.

Karkoulis, P.K.; Stravopodis, D.J.; Konstantakou, E.G.; Voutsinas, G.E. Targeted inhibition of heat shock protein 90 disrupts multiple oncogenic signaling pathways, thus inducing cell cycle arrest and programmed cell death in human urinary bladder cancer cell lines. Cancer Cell Int. 2013, 13, 11, doi:10.1186/1475-2867-13-11.

Kosmaoglou, M.; Kanuga, N.; Aguilà, M.; Garriga, P.; Cheetham, M.E. A dual role for EDEM1 in the processing of rod opsin. J. Cell Sci. 2009, 122, 4465–4472, doi:10.1242/jcs.055228.

Saeed, M.; Suzuki, R.; Watanabe, N.; Masaki, T.; Tomonaga, M.; Muhammad, A.; Kato, T.; Matsuura, Y.; Watanabe, H.; Wakita, T.; et al. Role of the Endoplasmic Reticulum-associated Degradation (ERAD) pathway in degradation of hepatitis C virus envelope proteins and production of virus particles. J. Biol. Chem. 2011, 286, 37264–37273, doi:10.1074/jbc.M111.259085.

Elfrink, H.L.; Zwart, R.; Baas, F.; Scheper, W. Inhibition of endoplasmic reticulum associated degradation reduces endoplasmic reticulum stress and alters lysosomal morphology and distribution. Mol. Cells 2013, 35, 291–297, doi:10.1007/s10059-013-2286-9.

Lin, Y.T.; Prendergast, J.; Grey, F. The host ubiquitin-dependent segregase VCP/p97 is required for the onset of human cytomegalovirus replication. PLoS Pathog. 2017, 13, e1006329, doi:10.1371/journal.ppat.1006329.

Bastola, P.; Wang, F.; Schaich, M.A.; Gan, T.; Freudenthal, B.D.; Chou, T.F.; Chien, J. Specific mutations in the D1–D2 linker region of VCP/p97 enhance ATPase activity and confer resistance to VCP inhibitors. Cell Death Discov. 2017, 3, 1–9, doi:10.1038/cddiscovery.2017.65.

Hili, D.S.; Martin, S.; Armstrong, J.L.; Flockhart, R.; Tonison, J.J.; Simpson, D.G.; Birch-Machin, M.A.; Redfern, C.P.F.; Lovat, P.E. Combining the endoplasmic reticulum stress-inducing agents bortezomib and fenretinide as a novel therapeutic strategy for metastatic melanoma. Clin. Cancer Res. 2009, 15, 1192–1198, doi:10.1158/1078-0432.CCR-08-2150.

Obeng, E.A.; Carlson, L.M.; Gutman, D.M.; Harrington, W.J.; Lee, K.P.; Boise, L.H. Proteasome inhibitors induce a terminal unfolded protein response in multiple myeloma cells. Blood 2006, 107, 4907–4916, doi:10.1182/blood-2005-08-3531.
